# Frequent Bullying Involvement and Brain Morphology in Children

**DOI:** 10.3389/fpsyt.2019.00696

**Published:** 2019-09-24

**Authors:** Ryan L. Muetzel, Rosa H. Mulder, Sander Lamballais, Andrea P. Cortes Hidalgo, Pauline Jansen, Berna Güroğlu, Meike W. Vernooiji, Manon Hillegers, Tonya White, Hanan El Marroun, Henning Tiemeier

**Affiliations:** ^1^Department of Child and Adolescent Psychiatry/Psychology, Erasmus MC University Medical Center, Rotterdam, Netherlands; ^2^Department of Epidemiology, Erasmus MC University Medical Center, Rotterdam, Netherlands; ^3^Institute of Education and Child Studies, Leiden University, Leiden, Netherlands; ^4^Generation R Study Group, Erasmus MC University Medical Center, Rotterdam, Netherlands; ^5^Department of Psychology, Education and Child Studies, Erasmus School of Social and Behavioral Sciences, Erasmus University Rotterdam, Rotterdam, Netherlands; ^6^Institute of Psychology, Leiden University, Leiden, Netherlands; ^7^Department of Radiology and Nuclear Medicine, Erasmus MC University Medical Center, Rotterdam, Netherlands; ^8^Department of Pediatrics, Erasmus MC University Medical Center, Rotterdam, Netherlands; ^9^The Department of Social and Behavioral Science, Harvard TH Chan School of Public Health, Boston, MA, United States

**Keywords:** cortical thickness, victimization, vertex-wise analysis, population based, fusiform

## Abstract

**Background:** Over the past few decades, bullying has been recognized as a considerable public health concern. Involvement in bullying is associated with poor long-term social and psychiatric outcomes for both perpetrators and targets of bullying. Despite this concerning prognosis, few studies have investigated possible neurobiological correlates of bullying involvement that may explain the long-term impact of bullying. Cortical thickness is ideally suited for examining deviations in typical brain development, as it has been shown to detect subtle differences in children with psychopathology. We tested associations between bullying involvement and cortical thickness using a large, population-based cohort.

**Methods:** The study sample consisted of 2,602 participants from the Generation R Study. When children were 8 years old, parents and teachers reported on common forms of child bullying involvement (physical, verbal, and relational). Questions ascertained whether a child was involved as a perpetrator (*n* = 82), a target of bullying (*n* = 92), as a combined perpetrator and target of bullying (*n* = 47), or uninvolved in frequent bullying (*n* = 2,381). High-resolution structural MRI was conducted when children were 10 years of age. Cortical thickness estimates across the cortical mantle were compared among groups.

**Results:** Children classified as frequent targets of bullying showed thicker cortex in the fusiform gyrus compared to those uninvolved in bullying (*B* = 0.108, *p*_corrected_ < 0.001). Results remained consistent when adjusted for socioeconomic factors, general intelligence, and psychiatric symptoms. Children classified as frequent perpetrators showed thinner cortex in the cuneus region; however, this association did not survive stringent correction for multiple testing. Lastly, no differences in cortical thickness were observed in perpetrator–targets.

**Discussion:** Bullying involvement in young children was associated with differential cortical morphology. Specifically, the fusiform gyrus, often involved in facial processing, showed thicker cortex in targets of frequent bullying. Longitudinal data are necessary to demonstrate the temporality of the underlying neurobiology associated with bullying involvement.

## Introduction

The past decades have witnessed bullying during childhood emerge as a considerable public health concern. Prevalence estimates are relatively high, although vary considerably by age, gender, frequency of involvement, and country (1). In addition to the immediate burden on the child, poor long-term outcomes have been consistently reported in those involved once they reach adulthood, including increased rates of psychiatric disorders, substance abuse, problems with social functioning, and suicidality (2–7). The persistence of these problems into adulthood suggests that a potential underlying neurobiological substrate may be linked to bullying involvement.

Bullying in children is formally characterized as unwanted, repeated, and aggressive behavior among peers which occurs in the context of an actual or perceived power imbalance (8). Involvement takes place in multiple forms, including physical (e.g., hitting, fighting), verbal (e.g., name calling, inappropriate comments), relational (e.g., social exclusion), and more. Those involved in bullying are often classified as being a bully (perpetrator), a victim (target of bullying), or involved in both forms as a perpetrator–target (9). Against the background of high prevalence estimates and the advent of cyber bullying, it is crucial to better understand bullying in the context of neural correlates, as such features could eventually help to predict and even explain the persistent psychosocial outcomes of bullying involvement (10).

*In vivo* structural brain imaging methods have proven effective in examining typical (11) and atypical morphological brain development (12) and are a promising tool for ascertaining any neural correlates of bullying involvement. Previous work has already shown how early-life adversities, such as abuse, early life stress, quality of maternal care, and growing up in institutional care, impact cortical and subcortical development in children (13–15). Despite this work demonstrating the sensitivity of structural neuroimaging to detect subtle morphological features of typical and atypical brain development, few studies have explored to what extent bullying involvement is associated with brain morphology and brain structure (16, 17). More substantial focus has been given to aspects of peer and social interaction using functional MRI (18) where, for example, anterior cingulate and prefrontal cortices have been implicated with differential functional activity in the context of exposure to social exclusion (19, 20). Recently, a large study of adolescents examined how structural brain volumes were related to peer victimization and psychopathology; changes in brain volumes which were related to peer victimization (specifically portions of the basal ganglia) were also predictive of internalizing problems later in life (21).

We aimed to study the association between bullying involvement and brain morphology in a large population-based setting. Parent- and teacher-rated bullying involvement was used to classify children as perpetrators, targets of bullying, or combined perpetrator–targets. We performed structural MRI to quantitatively assess the thickness of the cortical mantle, as well as hippocampal and amygdala volume; metrics shown to be associated with psychopathology and symptomatology in children. We hypothesized that targets of bullying involvement would display differences in cortical thickness in brain areas related to threat perception and sensitivity, fear, anxiety, emotional face processing, and emotional regulation (e.g., prefrontal cortex, cingulate gyrus, fusiform face area, and insula). We also hypothesized that perpetrators would differ in cortical thickness in areas related to emotional (dys)regulation (e.g., prefrontal cortex). Lastly, we hypothesized that those involved as perpetrator–targets would display the largest differences in cortical thickness in regions observed in both perpetrators and targets of bullying.

## Methods and Materials

### Participants

**Figure 1:**
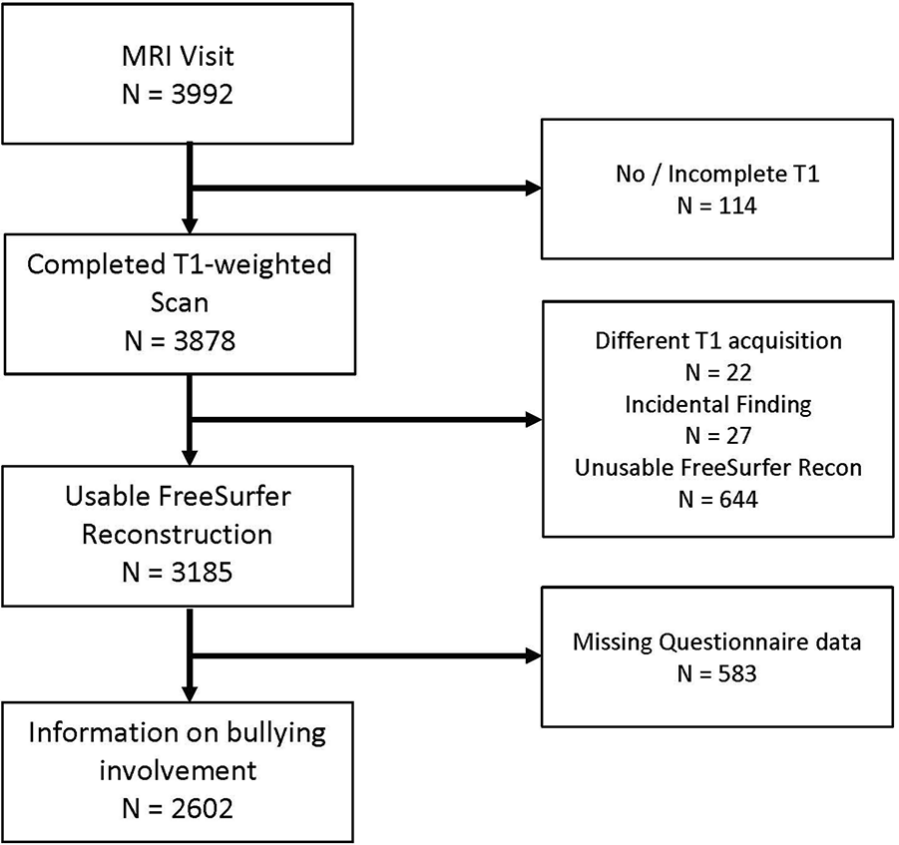
Flow chart indicating participant inclusion/exclusion from study population.

Participants in this study were part of the Generation R Study, a prospective prenatal birth cohort in Rotterdam, The Netherlands (22). When children were between the ages of 7 and 8, parents and teachers completed a questionnaire on children’s bullying involvement. At the age of 10, children visited our research-dedicated facility for a detailed behavioral assessment (23) and also underwent MRI (24). Of the 3,992 children who visited our MRI facility, 807 datasets were excluded due missing complete T_1_ scan (*n* = 114; 3%), a different T_1_ acquisition (*n* = 22, 0.6%), poor/insufficient data quality (*n* = 644; 16%), or incidental findings (*n* = 27; 0.7%, **Supplemental Table S1**). Of the remaining 3,185 children who had MRI data, 2,602 also had parent or teacher report information on bullying involvement and comprised the final study population. The flow chart depicted in **Figure 1** illustrates these exclusions in detail. The Medical Ethics Committee of the Erasmus Medical Center approved all study procedures, and all parents and children provided written informed consent and assent, respectively.

### Bullying Involvement Assessment

Three common forms of bullying involvement (9) were assessed using two separate informants: Physical, verbal, and relational bullying involvement were assessed by asking the child’s primary caregiver (most often the mother) and/or the child’s teacher. Separate questions ascertained whether the child was involved as a perpetrator or a target of bullying for each type of involvement (physical, verbal, relational involvement, for a total of six items). For example, parents and teachers were asked, “In the past few months, how often has your child been bullied by insults, being called names or being laughed at?” Full questions are presented in the Supplemental Material. Teachers were additionally asked whether a child was involved in material bullying. However, this item was not administered in the parent version of the questionnaire given the low endorsement rates by teachers and, therefore, was not used in the current analyses. Each item was rated on a 4-point scale ranging from “Never or less than once per month” to “More than twice per week” by the teachers and on a 5-point scale ranging from “Never” to “Several times per week” by the parents. Children were classified as perpetrators if their parent or their teacher indicated they physically, verbally, or relationally bullied other children once per week or more, which represents frequent involvement. In the event of disagreement between informants, children were classified as perpetrators if one informant indicated involvement (3, 25). Similarly, children were classified as targets of bullying if their parent or teacher indicated they were bullied by another child once per week or more. If a child was classified as both a perpetrator and as a target of bullying based on these criteria, they were reclassified as being involved as both, referred to as a perpetrator–target. Ratings from multiple informants were not available for all children, with roughly 33% receiving information from both informants, 53% receiving information from mothers only, and 14% receiving information from the teacher only. Cohen’s Kappa was in line with previous work, κ = 0.11, *p* < 0.05 (25–28). Although this agreement is relatively low, as highlighted by previous work (25), it is also consistent with agreement between informants in the context of behavioral and emotional problems (29).

### Image Acquisition

Neuroimaging data were collected on a study-dedicated, 3-Tesla MRI system (MR-750W, General Electric, Milwaukee, WI, US) using an eight-channel, receive-only head coil (24). Before scanning, children underwent a mock scanning session in order to familiarize them with the procedure and scanning environment. High-resolution, T_1_-weigthed structural MRI data were acquired using a coronal inversion recovery fast spoiled gradient recalled sequence with the following parameters: GE option BRAVO, T_R_ = 8.77 ms, T_E_ = 3.4 ms, T_I_ = 600 ms, flip angle = 10°, matrix size = 220 × 220, field of view = 220 mm × 220 mm, slice thickness = 1 mm, number of slices = 230, ARC acceleration factor = 2.

### Image Processing

Images were processed using the FreeSurfer version 6.0 analysis suite (30). First, DICOM data were converted to “MGZ” file format using the FreeSurfer “mri_convert” tool. The standard reconstruction was then conducted, where nonbrain tissue was removed, voxel intensities were corrected for B_1_ field inhomogeneities, voxels were segmented into white matter, gray matter, and cerebral spinal fluid, and surface-based models of white matter and gray matter were generated. Subcortical structures were automatically labeled, and volumes in cubic millimeter were extracted for the hippocampus and amygdala for this study. Cortical thickness was estimated at each point (vertex) along the cortical ribbon, and each point was also automatically assigned an anatomical label according to a predefined atlas (31). Thickness data for each participant were coregistered to a standard stereotaxic space and smoothed with a 10-mm full-width half-maximum Gaussian kernel. Cortical surface reconstructions were visually inspected for inaccuracies (32), and 16% of the scans were labeled as inadequate for data analyses.

### Covariates

Date of birth and sex were determined from medical records obtained at birth, and child ethnicity was defined based on the birth country of the parents. Maternal education level, a proxy for socioeconomic status, was assessed by questionnaire. Child nonverbal IQ was estimated using subtests from the Snijders–Oomen nonverbal intelligence test at the age-6 assessment (33). Lastly, child psychiatric symptoms were assessed using the parent-report Child Behavior Checklist (CBCL) administered at the age-10 assessment. The CBCL is a 100-item parental report of child behavioral and emotional problems which uses a Likert response format. The CBCL assesses a variety of domains, including internalizing (e.g., depressive/anxiety symptoms) and externalizing (e.g., attention problems). The square root transformed sum of all items (Total Problem Score) was utilized (34). For supplemental analyses (see statistical section below), additional covariates were tested for their impact on model estimates. First, for analyses involving targets of bullying, the CBCL Broadband Internalizing scale was used, as it focuses on emotional problems more common in this group. With the same rationale, the CBCL Broadband Externalizing scale was added in analyses of perpetrators of bullying. In order to rule out that childhood trauma explained any observed associations, exposure to physical and sexual abuse, derived from a retrospective parental-report of life events, was included. Briefly, a dichotomous (exposed/unexposed) variable was created if any of four items related to physical and sexual abuse were endorsed (35). Lastly, body mass index, estimated from height and weight measured at the age-6 assessment and normalized in accordance to Dutch growth curves for age and sex, was also included as a covariate in supplemental analyses.

### Statistical Analysis

Analyses were run using the R statistical software [version 3.4.3 (36)]. Multiple linear regression was used for analyses of hippocampal and amygdala volume. A custom in-house package was developed to run multiple linear regression at each cortical vertex (“QDECR,” https://github.com/slamballais/QDECR). A dichotomous variable for each of the three groups (perpetrator, target of bullying, and perpetrator–target) was created and reference coded to the individuals uninvolved in bullying. Regression analyses were run in three steps to adjust for potential confounding factors. All three models are presented in order to show the impact different confounding factors have on regression coefficients, with large changes in estimates indicative of the potential for residual confounding (remaining bias in estimates). Furthermore, as different neuroimaging studies often have limited data on various confounding factors, presenting analyses in this way allows for maximal comparison with existing/new literature, for example in the frequent case when only age and sex are available. Primary analyses were adjusted for age at MRI scan, sex, and child ethnicity (model 1). Additional analyses were run by further adjusting model 1 for maternal education and child nonverbal IQ (model 2). Lastly, to determine whether results were explained by child behavior problems, model 2 was additionally adjusted for child behavior problems (model 3).

In order to ascertain to what extent the perpetrator–target classification influenced results, sensitivity analyses were run where the perpetrator–target category was not considered. These children were dichotomously classified as targets of bullying or perpetrators. In further sensitivity analyses, continuous sum scores of bullying involvement were entered into regression models in order to complement categorical analyses. In order to determine whether observed associations were different between boys and girls, models were also run with a perpetrator group-by-sex interaction term. As internalizing problems are more often related to victimization and externalizing problems are more often related to perpetrator behavior, sensitivity analyses were run for model 3 where the CBCL total problems score was replaced with either the broadband internalizing score (targets of bullying) or the broadband externalizing score (perpetrators). Lastly, in order to rule out other potential confounding factors, exposure to traumatic events as well as body mass index were added to model 3 to ensure these factors did not account for any observed associations.

Given the large number of statistical tests, analyses were adjusted for multiple comparisons using Gaussian Monte Carlo simulations (37). Clusterwise *p* values were Bonferroni corrected for two hemispheres (*p* < 0.025), and, as it has shown high correspondence with actual permutation testing at the smoothing kernel used, a cluster-forming threshold (CFT) of *p* = 0.001 was selected for significance testing (38). As this threshold may be conservative, in line with genome-wide association studies, a “suggestive” yet still strict CFT was also employed (*p* = 0.005 CFT). For illustrative purposes, different CFTs are also displayed in figures and tables when a cluster was significant at the suggestive CFT 0.005 or below.

### Missing Data

Data were missing on covariates in a subset of participants for ethnicity, maternal education, nonverbal IQ and behavioral problems. In all cases, missingness was <11%. In order to retain the largest possible sample for linear regression analyses, these missing data were imputed utilizing the “mice” (multiple imputation by chained equations) package for multiple imputation (39). A number of variables that are correlated with these covariates were used in the imputation process. With 100 iterations, a total of 30 imputed datasets were generated, and results were pooled at each vertex using established methods (40).

### Nonresponse

Nonresponse was described with two sets of analyses: first, a comparison with children who participated in the age-6 assessment (roughly the age when the bullying assessment was conducted) but do not have MRI data at age 10 and, second, a comparison with children who participated in the MRI study but were excluded from analyses (e.g., due to poor data quality). Children who participated in the age-6 assessment but not in the current study had lower IQ (*M*_MRI_ = 104, *M*_nonresponder_ = 99, *p* < 0.05), higher total behavioral problem scores (*M*_MRI_ = 16.7, *M*_nonresponder_ = 18.5, *p* < 0.05), were less likely to be Dutch (*P*_MRI_ = 64%, *P*_nonresponder_ = 53%, *p* < 0.05), and their mothers were less likely to have acquired higher education (*P*_MRI_ = 63%, *P*_nonresponder_ = 52%, *p* < 0.05). Similarly, children who were excluded from the present study (e.g., because of motion artifact or missing a bullying assessment) tended to also have lower IQ (*M*_MRI_ = 104, *M*_excluded_ = 100, *p* < 0.05), higher total CBCL problem scores (*M*_MRI_ = 16.7, *M*_excluded_ = 18.3, *p* < 0.05), less likely to be Dutch (*P*_MRI_ = 64%, *P*_excluded_ = 52%, *p* < 0.05), and their mothers were less likely to have acquired higher education (*P*_MRI_ = 63%, *P*_excluded_ = 54%, *p* < 0.05).

## Results

Girls represented 51% of the sample, and children were on average 10.1 years (range, 8.5–11.9) old at the MRI visit. Based on parent report (mean age of child, 8.1 years; range, 7.5–9.9 years) and/or teacher report (mean age of child, 6.6 years; range, 4.6–9.6 years), 92 children (3.5%) were frequently involved as targets of bullying, 82 as perpetrators (3.2%), 47 as perpetrator–targets (1.8%), and 2,382 (91.5%) were uninvolved in frequent bullying. **Table 1** provides a detailed overview of the sample characteristics.

**Table 1:** Sample Characteristics.

	All	Target	Perpetrator	Perpetrator–target
	*N* = 2,602	*N* = 92	*N* = 82	*N* = 47
Age MRI	10.096 ± 0.57	10.052 ± 0.51	10.115 ± 0.61	10.098 ± 0.63
Girl, *N* (%)	1,325 (51)	39 (42)	22 (27)	19 (40)
IQ	103.66 ± 14.64	104.716 ± 16.41	100.706 ± 15.33	103.049 ± 15.88
Ethnicity, *N* (%)*				
Dutch	1,655 (64)	59 (64)	39 (49)	27 (59)
Other Western	232 (9)	12 (13)	2 (2)	3 (6)
Non-Western	693 (27)	21 (23)	39 (49)	16 (35)
Maternal Education, *N* (%)*				
Primary/Secondary	879 (37)	29 (33)	38 (55)	15 (36)
Higher	1,524 (63)	58 (67)	31 (45)	27 (64)

Note: Values represent mean ± standard deviation unless otherwise noted. *Owing to missing data, some cells do not sum to complete sample size.

### Targets of Bullying

**Figure 2:**
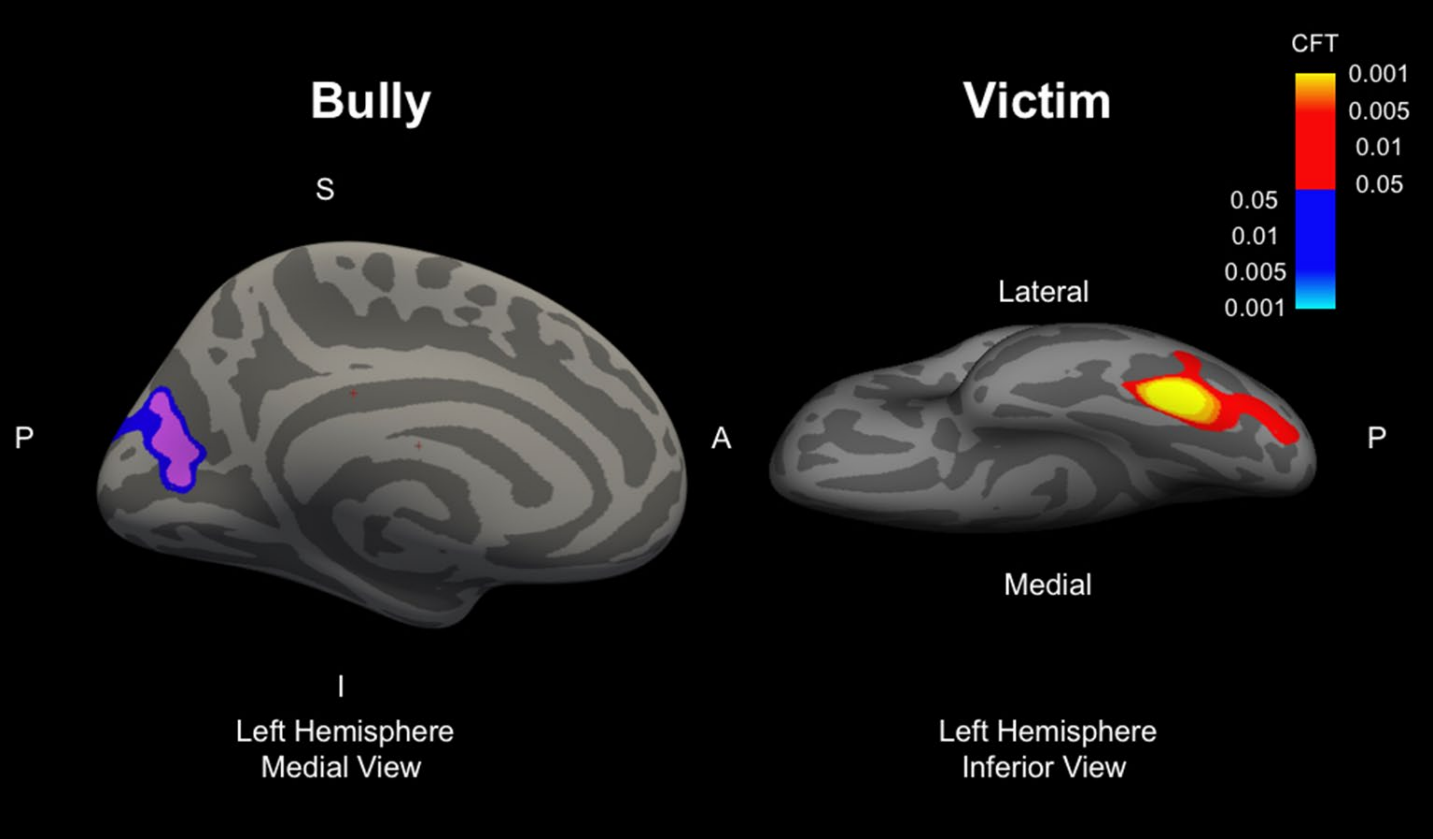
Images represent the left hemisphere clusters for perpetrators (left panel, view from medial side of the brain) and targets of bullying (right panel, view from inferior side of brain). Clusters represent areas which are different from children uninvolved in bullying (reference group). Models included one term for each of the three groups (perpetrators, targets, perpetrator–targets) all in the same model, and all reference coded to those uninvolved in bullying. S, superior, P, posterior, A, anterior, I, inferior, CFT, cluster-forming threshold. Red–yellow colors refer to thicker cortex, blue–light blue colors refer to thinner cortex.

Whole-brain vertex-wise analyses of cortical thickness showed that children identified as targets of bullying had thicker cortex in the left fusiform gyrus compared to those uninvolved in frequent bullying (**Figure 2**, **Table 2**). Results remained highly consistent across model 1 (*B* = 0.107, SE = 0.027, size = 312 mm^2^, *p*_CFT_ = 0.001, adjusted for age, sex, and ethnicity), model 2 (*B* = 0.108, SE = 0.027, size = 312 mm^2^, *p*_CFT_ = 0.001, additionally adjusted for child IQ and maternal education level), and model 3 (*B* = 0.110, size = 290 mm^2^, *p*_CFT_ = 0.001, additionally adjusted for child behavioral problems), suggesting minimal residual confounding through various categories of covariates (**Table 2**). Results remained highly consistent when additionally adjusting model 3 using the broadband internalizing scale rather than the total problems scale (*B* = 0.107, SE = 0.027, size = 307 mm^2^, *p*_CFT_ = 0.001). In addition, adjusting model 3 for exposure to traumatic life events (*B* = 0.107, SE = 0.027, size = 295 mm^2^, *p*_CFT_ = 0.001) or for body mass index (*B* = 0.108, SE = 0.027, size = 279 mm^2^, *p*_CFT_ = 0.001) did not change the results (**Supplemental Table S2**). A sex-by-target of bullying interaction term showed no significant clusters, suggesting the association is similar in boys and girls.

**Table 2:** Results from whole-brain cortical thickness analyses.

Group	Model	MNI_X_	MNI_Y_	MNI_Z_	CFT	B	SE	*N* Vertices	Area (mm^2^)
Target	1	−40.5	−54.8	−20.3	0.05	0.076	0.027	2,073	1,404
					0.01	0.093	0.026	839	550
					0.005	0.098	0.027	696	451
					0.001	0.107	0.027	488	312
	2	−40.5	−54.8	−20.3	0.05	0.076	0.027	2,083	1,412
					0.01	0.093	0.026	848	556
					0.005	0.099	0.026	702	456
					0.001	0.108	0.027	488	312
	3	−40.7	−53.9	−20.2	0.05	0.076	0.027	2091	1423
					0.01	0.094	0.026	823	539
					0.005	0.100	0.027	677	439
					0.001	0.110	0.027	455	290
Perpetrator	1	−14.8	−69.5	15.3	0.05	−0.067	0.027	2,206	1,418
					0.01	−0.074	0.026	1,139	689
					0.005	−0.077	0.027	840	501
	2	−14.8	−70	15.6	0.05	−0.072	0.027	2,158	1,389
					0.01	−0.081	0.026	1,030	615
					0.005	−0.084	0.027	735	435
	3	−14.8	−70	15.6	0.05	−0.072	0.027	2,004	1,292
					0.01	−0.081	0.027	839	496

Model 1 is adjusted for age at MRI, sex, and ethnicity. Model 2 is additionally adjusted for child IQ and maternal educational level. Model 3 is additionally adjusted for child psychiatric symptoms. MNI, Montreal Neurological Institute Coordinates, CFT, cluster-forming threshold for correction for multiple comparisons, B, unstandardized regression coefficient, N Vertices, number of vertices in cluster, Area, surface area of cluster.

In additional analyses utilizing a two-group (perpetrator or target of bullying) classification, results remained unchanged, suggesting the smaller perpetrator–target category did not influence results. When using a continuous sum score of victimization rather than categorical groupings, a similar cluster appeared in the fusiform gyrus, where high scores on victimization were related to thicker cortex (*p*_uncorrected_ = 0.0001, **Supplementary Figure S1**). However, this result did not remain after correction for the stringent multiple comparisons threshold. Lastly, no difference was observed in hippocampal or amygdala volume.

### Perpetrators

Children classified as perpetrators showed a thinner cortex in the cuneus at the “suggestive” threshold after correcting for multiple comparisons (*p*_CFT_ = 0.005) but not at the more stringent threshold (*p*_CFT_ = 0.001) and not fully adjusted for all covariates (i.e., model 3). Results remained consistent across the basic (model 1, *B* = −0.077, size = 501 mm^2^, *p*_CFT_ = 0.005) and adjusted model (model 2, *B* = −0.084, size = 435 mm^2^, *p*_CFT_ = 0.005), although disappeared when adjusting for child behavioral problems (**Table 2**). Additional analyses using a two-group classification (i.e., omitting the perpetrator–target category) showed consistent results. However, the cuneus cluster was not present when bullying involvement was examined continuously. Lastly, no difference was observed in hippocampal or amygdala volume.

### Perpetrator–Targets

No differences in cortical thickness were observed between perpetrator–targets and those uninvolved in bullying after correcting for multiple comparisons. Furthermore, no difference was observed in hippocampal or amygdala volume.

## Discussion

This large population-based study demonstrates differences in cortical morphology in children involved in bullying. Specifically, children identified as targets of bullying showed thicker cortex in the fusiform region compared to children uninvolved in bullying. The results demonstrate a new link between bullying involvement and structural brain morphology. Importantly, the results also provide an integral starting point for future work examining how cortical brain morphology relates to the persistent social and mental health problems that accompany those involved in bullying.

Children who were frequently victimized by perpetrators showed thicker cortex in the fusiform gyrus. This area, also part of Brodmann area 37, has been implicated in a wide array of functions, including facial and emotion processing, language, and theory of mind. Thicker cortex in this region could therefore be related to how targets of bullying perceive or recognize the faces of their aggressors. Interestingly, individuals with social anxiety disorder have been shown to exhibit differential neural activity to fearful as well as threatening faces (41, 42). A similar extension could be drawn to targets of bullying, where a sensitivity to certain facial expressions (e.g., angry/aggressive) could develop as a consequence of bullying. Alternatively, language ability has previously been proposed as a potential risk factor for targets of bullying, where children with underdeveloped language skills have been shown to be bullied more often (43, 44). As the fusiform gyrus has been implicated in aspects of verbal fluency (45), it is also possible that thicker cortex here represents a delayed development of language ability, which could in turn translate into a risk factor for bullying. Importantly, classification of targets of bullying was defined as being bullied once per week or more, which denotes frequent bullying involvement. When victimization was treated continuously rather than categorically, a similar cluster was observed, which suggests that such features of the fusiform may track into less frequently bullied children. Although the fusiform gyrus has been implicated in psychopathology (41), the current study was not able to determine whether it plays a mediating role in the development of psychopathology, as recent work has shown with other brain regions (21). Importantly, brain morphology linked to involvement in bullying may later manifest in other, more distant brain regions through atypical development of functional connectivity; such a downstream pathway may instead explain the persistent mental health and social problems experienced later in life.

At a conservative threshold for multiple testing correction, no differences were observed in cortical thickness in those classified as perpetrators. However, at a “suggestive” *p* value threshold and in models not adjusted for total psychiatric problems, a thinner cortex in the cuneus area was observed in those identified as perpetrators compared to those uninvolved in bullying. Part of the occipital lobe, the cuneus, is involved in various aspects of visual processing. A section of the cuneus has also often been implicated in the default mode network, one of the most commonly derived networks in resting-state functional MRI. Thus, future efforts should explore to what extent the default mode network is implicated in those involved in bullying. However, importantly, as this association was not significant in analyses where bullying was quantified continuously and was only observed at a relaxed correction for multiple testing, these results should be interpreted cautiously in the absence of external replication.

Interestingly, in children identified to be involved as perpetrator–targets, no differences were observed in cortical thickness. Given this group of children has the overall poorest prognosis, with higher rates of psychopathology and other problems later in life (25, 46, 47), this lack of difference in brain morphology is contrary to our *a prior* hypothesis. One potential explanation may lie in the sample size of this subgroup; it was the smallest group, with only 47 children, potentially limiting our ability to detect any differences. Conversely, there is likely considerable heterogeneity in any underlying morphological features in this group, suggesting that subtypes of bullying involvement may be important for brain development, or that other analytical methods may be necessary to detect differences (48).

Brain morphology has also been studied in the context of early life stress, trauma, maltreatment, and other experiences (49–53). Differences across studies in terms of findings, methods used, and populations examined make summarizing the literature a challenge, although some interesting patterns emerge. Associations with the amygdala and hippocampus are certainly a common theme, although findings have been inconclusive (50, 54–56). One central theory that aims to explain such deviations in brain development revolves around chronic stress exposure (57), and given the density of stress hormone receptors in the hippocampus, it may be particularly sensitive. Interestingly, no differences were found in this study in children who were targets of bullying. Timing of early-life exposures may be of particular relevance (14), which could explain why no association was found. Specifically, children may have been exposed to bullying behavior at varying times and durations, leading to heterogenous changes limbic brain structures that are difficult to detect, or may emerge later in life.

One important consideration of this study is the temporality of the brain–behavior relationship. As this study is based on a single neuroimaging assessment, it is not possible to delineate whether differences observed in cortical thickness develop before or after children become involved in bullying. In the context of targets of bullying, both scenarios are plausible. Targets of bullying could show differential fusiform development over time, either as an adverse consequence of bullying or even as a compensatory mechanism resulting from exposure to such behavior. Alternatively, such features of the fusiform gyrus could be present before the exposure to bullying, potentially acting as a predisposing factor. An example of such a mechanism can be found in the preceding paragraph discussing language ability; children with poor language abilities may be more prone to exposure to bullying. Future studies with longitudinal designs will allow for the determination of where on the neurodevelopmental trajectory they lie.

The established link between bullying involvement and persistent social and psychiatric problems later in life suggests the potential for a related and underlying neurobiological substrate. A similar construct has been proposed in the context of child maltreatment (10). Recent work has shown evidence for such a link *via* the basal ganglia (21). Alterations in the fusiform that potentially result from bullying could explain some facets of a given psychiatric disorder, for example altered cortical activity in individuals with anxiety disorder in response to emotional face processing (41) or emotionally valent images in individuals with depression (58). Importantly, brain alterations related to bullying involvement during childhood, which eventually co-occur with psychiatric sequela later in life, may require special consideration in future brain imaging research; the underlying neurobiology may be unique to bullying involvement and not necessarily common or etiological to the psychiatric symptomatology or overarching disorder. Such a phenomenon of an early life adversity leading to a particular brain alteration which co-occurs with psychiatric symptoms could explain some of the heterogeneity in the psychiatric neuroimaging literature and thus the lack of robust imaging biomarkers (59, 60).

Utilizing one of the world’s largest pediatric neuroimaging cohorts, we were able to examine the structural neural correlates of bullying involvement. Accompanying the power from this large sample size is the improved generalizability of the findings resulting from the population-based sampling, both in the reference group (those not involved in bullying) as well as in the groups exposed to bullying. Given the prospective and broad nature of the cohort, crucial information on potential confounding factors was also available. However, despite these clear strengths, some limitations warrant discussion. First, as described above, this study lacks repeated measurements of both bullying and brain imaging, and the assessment of bullying involvement takes place at a different age (i.e., before) than the MRI assessment. Longitudinal data will be crucial in delineating the precise temporal sequence of events and offer a crucial developmental perspective. Also of important note is the nonresponse analysis, which showed that the subsample children included in this study on average had slightly different characteristics compared with the full sample (e.g., 4 IQ points higher), suggesting some selection effects. Another broad issue in research on bullying involvement is related to how the data are characterized (e.g., continuous vs. categorically, frequency of involvement, etc.), which may impact results. Future work may also continue to explore latent constructs or latent classes of bullying involvement, which may offer additional insight by data-driven incorporation information (21). Lastly, this study relied on parent- and teacher-reported measures of bullying involvement, rather than child self-reports. Although parents and teachers have been shown to be reliable informants of bullying involvement, other strategies, such as peer nomination (61) and self-reporting, likely provide information with added value on bullying involvement, as victimization has been shown to be underreported in the absence of self-report (62).

This study demonstrates a link between bullying involvement and brain morphology in school-age children. In particular, children who are victimized by perpetrators have thicker cortex when compared to those uninvolved in bullying. These data offer evidence of disrupted cortical morphology in those involved in bullying and may offer cues to future work investigating the neurobiological underpinnings of associated and persistent problems later in life. Future work should utilize longitudinal neuroimaging data to concretely ascertain the different developmental trajectories involved.

## Data Availability

The datasets generated and/or analyzed during the current study are not publicly available due to legal and ethical regulations, but may be made available upon request to the Director of the Generation R Study, Vincent Jaddoe (, v.jaddoe@erasmusmc.nl), in accordance with the local, national, and European Union regulations.

## Ethics Statement

The Medical Ethics Committee of the Erasmus Medical Center approved all study procedures, and all parents and children provided written informed consent and assent, respectively.

## Author Contributions

RLM and HT developed the project and drafted the manuscript. RHM and SL collected data. RLM, RHM, SL, AC, TW, and HM conducted quality assurance of the data. RLM and SL conducted statistical analyses and developed software. RHM, SL, AC, PJ, BG, MV, MH, TW, and HM interpreted results and helped to draft and critically revise the manuscript. HT oversaw all aspects of the project.

## Funding

This study was supported by the Sophia Foundation project S18-20 (awarded to R.L.M.). The Netherlands Organization for Health Research and Development (ZonMw) Vici project 016.VICI.170.200 (awarded to H.T.), and the Dutch Research Agenda (NWA) NeuroLabNL (400.17.602). Supercomputing resources were supported by the NWO Physical Sciences Division (Exacte Wetenschappen) and SURFsara (Cartesius compute cluster, www.surfsara.nl). HM was supported by the European Union’s Horizon 2020 research and innovation program (grant agreement 633595 and 733206). APCH was supported by the Netherlands Organization for Scientific Research. The general design of Generation R Study is made possible by financial support from the Erasmus Medical Center, Rotterdam, the Erasmus University Rotterdam, ZonMw, The Netherlands Organization for Scientific Research (NWO), and the Ministry of Health, Welfare, and Sport.

## Conflict of Interest Statement

The authors declare that the research was conducted in the absence of any commercial or financial relationships that could be construed as a potential conflict of interest.
