# Supplementary material for: Frequent Bullying Involvement and Brain Morphology in Children
Source: Front Psychiatry. 2019 Sep 24;10:696. doi: 10.3389/fpsyt.2019.00696 (PMC6771170; doi:10.3389/fpsyt.2019.00696)
Supplement: Supplementary file 1 [file DataSheet_1.docx]

**Supplemental Material**

**Supplemental Table S1:** Excluded cases due to incidental findings

| Incidental Finding | Frequency |
| --- | --- |
| Cerebral or Cerebellar Cyst | 18 |
| Dysgenesis of the Corpus Callosum | 2 |
| Ventriculomegaly | 2 |
| Ependymoma | 1 |
| Glioma | 1 |
| Prior Neurosurgery | 1 |
| Radiologically Isolated Syndrome | 1 |
| Gray Matter Heterotopia | 1 |

**Supplemental Table S2** Comparison of fusiform cluster across different models in targets of bullying

| Model | B | SE | N Vertices | Area (mm^2^) |
| --- | --- | --- | --- | --- |
| 1 | 0.107 | 0.027 | 488 | 312 |
| 2 | 0.108 | 0.027 | 488 | 312 |
| 3 | 0.110 | 0.027 | 455 | 290 |
| Internalizing | 0.107 | 0.027 | 482 | 307 |
| Body Mass Index | 0.107 | 0.027 | 437 | 279 |
| Traumatic Events | 0.106 | 0.027 | 461 | 295 |

Note: Model 1 is adjusted for age at MRI, sex, and ethnicity. Model 2 is additionally adjusted for child IQ and maternal educational level. Model 3 is additionally adjusted for child psychiatric symptoms. Internalizing indicates Model 2 + CBCL Broadband Internalizing problems, Body Mass Index = Model 3 + Body Mass Index, and Traumatic Events = Model 3 + Traumatic Events (present/ not present). All results presented for the p < 0.001 cluster forming threshold.

**Supplemental Figure S1. Continuous victimization score association with cortical thickness**

**
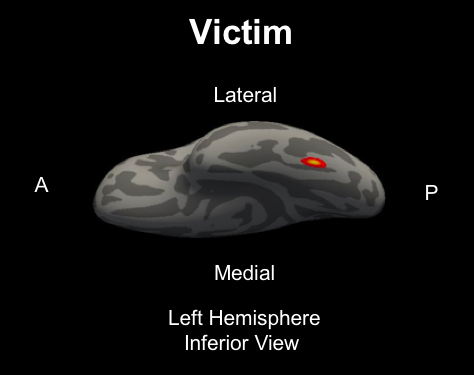
**

Inferior view of the lateral hemisphere. A=Anterior, P=Posterior. Red-yellow color indicates region of cortical thickness associated with continuous victimization score p< 0.0001 uncorrected for multiple testing.

**Methods**

Bullying involvement assessment

Questions used for the bullying involvement assessment included the following:

Parent report: Target of bullying

*1.) In the past few months, how often has your child been bullied by insults, name calling, or being laughed at?*

*2.) In the past few months, how often has your child been bullied by way of spitting, hitting, kicking, or pinching?*

*3.) In the past few months, how often has your child been: bullied by being excluded from activities, ignored by other children or gossiped about?*

Parent report: Perpetrator of bullying

*1.) In the past few months, how often has your child bullied other children by saying insulting things to them, calling them names or laughing at them?*

*2.) In the past few months, how often has your child bullied other children by spitting at them, hitting, kicking, or pinching them?*

*3.) In the past few months, how often has your child bullied other children by excluding them from activities, ignoring them, or by gossiping about them?*

Parent-rated questions could be answered with the following options:

*Never, seldom (1 or 2 times), 2-to-3 times per month, 1 time per week, and several times per week.*

Teacher report: Target of bullying

*1.) In the last 3 months, how often has the child been verbally bullied (laughed at, insulted, or teased).*

*2.) In the last 3 months, how often has the child been physically bullied (hit, kicked, pinched, or bitten).*

*3.) In the last 3 months, how often has the child been excluded?*

Teacher report: Perpetrator of bullying

*1.) In the last 3 months, how often has the child verbally bullied other children (laughed at, insulted, or teased).*

*2.) In the last 3 months, how often has the child physically bullied other children (hit, kicked, pinched, or bitten).*

*3.) In the last 3 months, how often has the child excluded other children?*

Teacher-rated questions could be answered with the following options:

*Never / almost never, 1-to-3 times per month, 1-to-2 times per week, more than 2 times per week.*
